# Anthocyanins in Floral Colors: Biosynthesis and Regulation in Chrysanthemum Flowers

**DOI:** 10.3390/ijms21186537

**Published:** 2020-09-07

**Authors:** Manjulatha Mekapogu, Bala Murali Krishna Vasamsetti, Oh-Keun Kwon, Myung-Suk Ahn, Sun-Hyung Lim, Jae-A Jung

**Affiliations:** 1 Floriculture Research Division, National Institute of Horticultural & Herbal Science, Rural Development Administration, Wanju 55365, Korea; manjubio7@gmail.com (M.M.); kok5510@korea.kr (O.-K.K.); ahnms@korea.kr (M.-S.A.); 2 Chemical Safety Division, National Institute of Agricultural Sciences, Rural Development Administration, Wanju 55365, Korea; vbmk84@gmail.com; 3 Division of Horticultural Biotechnology, School of Biotechnology, Hankyoung National University, Anseong 17579, Korea; limsh2@hknu.ac.kr

**Keywords:** breeding, genetic manipulation, ornamental plants, petal color, plant pigments

## Abstract

Chrysanthemum (*Chrysanthemum morifolium*) is an economically important ornamental crop across the globe. As floral color is the major factor determining customer selection, manipulation of floral color has been a major objective for breeders. Anthocyanins are one of the main pigments contributing to a broad variety of colors in the ray florets of chrysanthemum. Manipulating petal pigments has resulted in the development of a vast range of floral colors. Although the candidate genes involved in anthocyanin biosynthesis have been well studied, the genetic and transcriptional control of floral color remains unclear. Despite advances in multi-omics technology, these methods remain in their infancy in chrysanthemum, owing to its large complex genome and hexaploidy. Hence, there is a need to further elucidate and better understand the genetic and molecular regulatory mechanisms in chrysanthemum, which can provide a basis for future advances in breeding for novel and diverse floral colors in this commercially beneficial crop. Therefore, this review describes the significance of anthocyanins in chrysanthemum flowers, and the mechanism of anthocyanin biosynthesis under genetic and environmental factors, providing insight into the development of novel colored ray florets. Genetic and molecular regulatory mechanisms that control anthocyanin biosynthesis and the various breeding efforts to modify floral color in chrysanthemum are detailed.

## 1. Introduction

Flowers are beautiful and vital organs, which provide eye-catching attributes to plants with their varied colors and morphology. Among flower attributes, petal color is economically and biologically important [1,2,3]. Floral color greatly influences customer choice of cut flowers, potted flowers, and garden flowers. Floral hue also plays a key role in attracting pollinators for reproduction. In addition to attracting pollinators, pigments protect against photo-oxidative damage, and provide resistance to biotic and abiotic stresses [4]. Therefore, the manipulation of floral color is an important goal for ornamental plant breeders.

The global cut flower market is dominated by major ornamental flowers, including rose, chrysanthemum, carnation, lily, and gerbera, which have commercial value and provide a high yield. Chrysanthemum is one of the most popular ornamental plants and is the second most economically important cut flower in the global market [5]. The quality of chrysanthemum flowers is evaluated based on its color, and hence color is important for the determination of commercial value [6,7]. The compound inflorescence of the chrysanthemum flower has central disc florets, which are usually yellow or green, whereas the marginal ray florets present a wide range of colors, including purple, green, red, pink, orange, and white [8]. Although floral color in wild chrysanthemum is limited to yellow, white, and pink, advances in chrysanthemum breeding have enabled the development of a broad spectrum of floral colors, including red, orange, dark red, and green.

Floral color intensity is mainly due to the accumulation of a single pigment or a combination of pigments, and flowers with no pigments have also been reported [8]. Plant biproducts, classified broadly as carotenoids and flavonoids, underly the colorful pigmentation of flower petals [9]. Carotenoids, classified as carotenes and xanthophylls, are the most widely distributed pigments, and are cyclization-produced organic molecules of a C40 polyene backbone with an ionone ring [10]. Carotenoids can impart yellow, orange, and bright red colors and their specific compositions vary widely in the petals of ornamental flowers. Flavonoids are the largest class of secondary metabolites that are widely distributed in the plant kingdom [11,12]. Flavonoids are characterized by two phenyl rings, and a heterocyclic C_15_ carbon ring as the backbone. Based on the specific structure and the functional group present, flavonoids can be divided into several groups: chalcones, flavanones, flavones, flavanonols, flavonols, flavanols, and anthocyanins. This widespread and important group of pigments contributes to a wide spectrum of petal colors, from pale yellow to orange, red, blue, purple, and magenta. In particular, anthocyanins are the largest group of pigments among flavonoids, which impart diverse colors in flower, from orange to red and from purple to blue [13]. Major pigments that impart floral colors are listed in Table 1. Chrysanthemum cultivars are considered a good source of anthocyanin for commercial-level extraction, since they contain numerous flowers in a single plant and a wide range of floral colors [14] Therefore, a deeper understanding of the molecular mechanism underlying anthocyanin regulation that influences petal color formation would facilitate more efficient breeding efforts to generate novel colors and patterns in chrysanthemum flowers. Thus, this review focuses on ray petal color formation in chrysanthemum, particularly that mediated by anthocyanin biosynthesis. The role of anthocyanins in plant pollinator attraction and stress tolerance is discussed. This review also provides a brief and cumulative overview of recent progresses in the study of genetic and environmental regulatory factors that influence anthocyanin biosynthesis. Moreover, a summary on the breeding efforts aiming at modifying and developing novel floral hues in chrysanthemum is presented.

## 2. Anthocyanins in Pollination Ecology

Floral color and scent are the traits that attract pollinators. Floral color acts as a sensory signal by endorsing the quality and quantity of floral rewards [15]. Pollinators exhibit a natural preference towards specific floral attributes, which they associate with the flower that provides food sources such as nectar and pollen [16]. Floral color provides flowers with radiance and attractiveness, which can act as a visualization signal to the pollinators, increasing the number of pollinator visits and thereby increasing the pollinator foraging efficiency and reproductive success. In some cases, flowers change their color, allowing the pollinators to avoid unrewarding and old flowers and to select rewarding flowers. This reduces repeated visits to the same flower and thus increase the pollination efficiency of the visitors [17,18]. A specific class of pollinators is usually associated with specific floral colors, which might be dependent on the perception of the pollinators. The majority of insect pollinators possess photoreceptors, which detect the UV part of the light spectrum as well as the visible spectrum [19,20]. UV light is reflected by the flowers of angiosperms [21]. UV-absorbing nectar guides aid the insects to rightly orient to obtain the nectar [22]. These UV patterns on the flower help the proper landing and foraging of the pollinator [23]. Attempts have been made to assess pollinator discrimination towards different colored petals in flowers. Notably, flowers with different pigments are attractive to different classes of pollinators. Bradshaw and Schemske (2003) [24] showed that bumblebees and hummingbirds clearly distinguished the near-isogenic lines of *Mimulus*, which only differed in floral color. Plants of *Mimulus lewisii*, which possess a pink flower due to the accumulation of anthocyanins, are primarily pollinated by bumblebees, while *Mimulus cardinalis*, characterized by orange/red colored flowers due to the combined accumulation of anthocyanins and carotenoids, are pollinated by hummingbirds. Additionally, when the yellow upper (YUP) locus, which is responsible for carotenoid accumulation was introgressed from each of these Mimulus species into other background, it resulted in orange-colored *M. lewisii* flowers and deep pink-colored *M. cardinalis* flowers. Orange *M. lewisii* flowers were then visited by more hummingbirds than bumblebees, and the pink *M. cardinalis* flowers were visited by more bumblebees than hummingbirds. Hence, it is likely that floral color including anthocyanin accumulation, plays a major role in the attraction of specific pollinators.

## 3. Anthocyanins in Stress Tolerance

Apart from the floral parts, anthocyanins often occur transiently in the root, stem, and leaf tissues of plants at specific developmental stages in response to environmental factors, including UV radiation, cold temperatures, and water stress. Although the biosynthesis and molecular control of anthocyanin in the floral parts have been well studied, the adaptive advantages of anthocyanin accumulation in non-reproductive tissues are unclear. The environmental induction of anthocyanin accumulation in the vegetative tissues reflects the response of the plant to environmental factors, including biotic and abiotic stresses [25]. Different stresses induce different kinds of anthocyanins with a change in the pattern of decoration, suggesting that these anthocyanins have unique purposes during stress. Arabidopsis grown under different abiotic stress conditions was found to accumulate high levels of total anthocyanins, with each stress condition inducing a different kind of anthocyanin [26]. Overexpression of *RsMYB1*, a positive regulator of anthocyanin biosynthesis, was found to induce the expression of genes related to metal detoxification in addition to enhanced anthocyanin accumulation in petunia, suggesting an important role of anthocyanins in metal detoxification in horticultural crops [27]. Furthermore, increased anthocyanin levels enabled petunia plants overexpressing the *RsMYB1* gene to tolerate low pH stress [28]. In chrysanthemum, R2R3-MYB transcription factors, which are involved in the regulation of anthocyanin biosynthesis, have also been linked to abiotic stress tolerance. Heterologous expression of *CmMYB2* was found to enhance the salinity and drought stress tolerance of *Arabidopsis*. *CmMYB2* increased the efficient ABA synthesis and more effective ABA signaling. Collectively, these processes induced the higher expression levels of some key abiotic stress responsive genes in transgenic *Arabidopsis*. Hence, *CmMYB2* is proposed as a promising candidate gene for abiotic stress tolerance in chrysanthemum [29].

## 4. Anthocyanin Biosynthesis in Chrysanthemum Flowers

**Figure 1:**
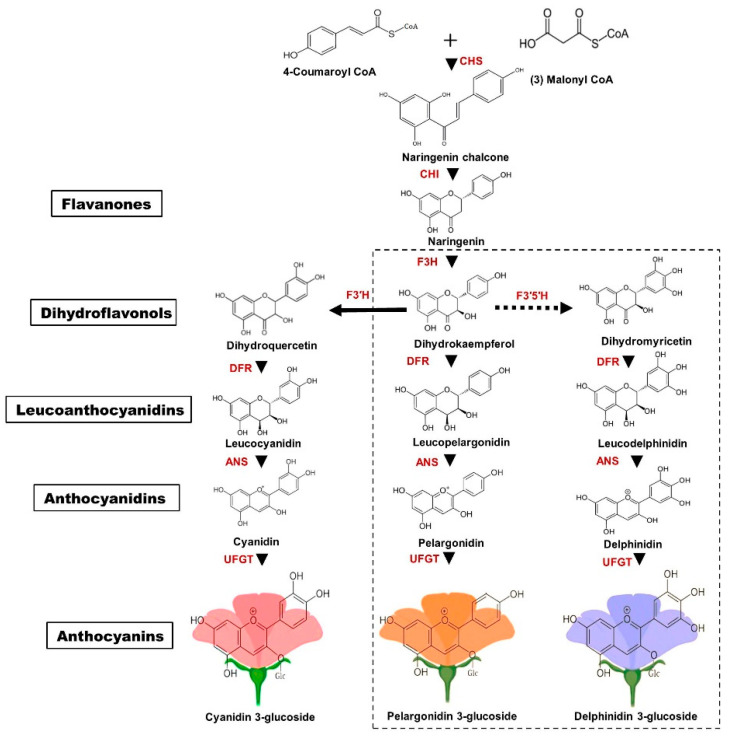
Schematic representation of the anthocyanin biosynthesis pathway. Enzymes involved in the biosynthesis pathway are shown in red. CHS—chalcone synthase; CHI—chalcone isomerase; F3H—flavanone 3-hydroxylase; F3′H—flavonoid 3′-hydroxylase; F3′5′H—flavonoid 3′5′- hydroxylase; DFR—dihydroflavonol 4-reductase; ANS—anthocyanidin synthase; UFGT— UDP-glycosyl transferase. Chrysanthemum flowers only accumulate cyanidin-based anthocyanins; therefore, the cyanidin pathway is indicated by a solid arrow line. The dotted box indicates pelargonidin and delphinidin synthesis, which are naturally absent in chrysanthemum petals. The colored anthocyanin background represents the respective color attributed by that particular compound.

Anthocyanins are plant secondary metabolites that have been well characterized in terms of their chemistry and structure [30]. These water-soluble pigments are localized in various organs, including leaves, flowers, and fruit, and are the main pigments that impart pink to purple-red colors in chrysanthemum ray petals, whereas a lack of their accumulation results in white ray florets [31,32]. Anthocyanins are the sugar-containing counterparts (3-glucosides) of anthocyanidins. They possess a characteristic C_6_-C_3_-C_6_ carbon skeleton and can be subdivided into anthocyanidin sugar-free aglycones and anthocyanin glycosides. Anthocyanidins consist of two aromatic benzene rings separated by an oxygenated heterocycle [33]. Of the 20 anthocyanidins identified, only six are common in plants [34]. Determination of flower petal color is largely dependent on the number of hydroxyl groups present in the skeletal B-ring, and the color shifts towards blue as the number of hydroxyl groups increases [33]. Anthocyanin color has been associated with changes in sugar moieties and organic acids in anthocyanidins, which form anthocyanins in a species-specific manner [9]. Generally, these anthocyanidin pigments accumulate as glycosylated forms called anthocyanins, since anthocyanidins are inherently more unstable. Structurally, these anthocyanins are characterized by carbon 3 linked through oxygen to a sugar residue, which is more frequently glucose, and also with other sugar moieties like galactose, xylose, rhamnose, and arabinose. The most prevalent anthocyanidins distributed in flowers are cyanidin, delphinidin, and pelargonidin. Of these three basic groups of anthocyanins, ray florets of chrysanthemum mainly accumulate cyanidin-based anthocyanins, such as cyanidin-3-*O*- (6″-*O*-malonyl) glucoside and cyanidin 3-*O*-(3″6″-di-*O*-malonyl) glucoside [8,35,36,37]. Early reports suggested that the pink to purple-red color in cyanidin-based ray petals depends on the cyanidin content [38]. Notably, chrysanthemums do not naturally accumulate delphinidin-based anthocyanins [39]. However, transgenic studies in the past decade have produced purple/violet transgenic chrysanthemum flowers, likely to be due to the accumulation of delphinidin-based anthocyanins, including delphinidin 3-(6″-malonyl) glucoside and delphinidin 3-(3″,6″-dimalonyl) glucoside in flower petals [39]. The generation of blue-colored chrysanthemums is further discussed in Section 8.5. Recently, transgenic chrysanthemum with novel blue petals has been generated, with the accumulation of delphinidin-based anthocyanin, delphinidin 3-*O*-(6″-*O*-malonyl) glucoside-3′,5′-di-*O*-glucoside, and co-pigmentation found in blue petals [40]. Anthocyanin biosynthesis is conserved across species; however, the mechanism of anthocyanin degradation remains unclear. [33,41,42]. Generally, anthocyanin biosynthesis involves sub-sequential metabolic pathways, including one molecule of 4-coumaroyl CoA, derived from the general phenylpropanoid pathway and three molecules of malonyl CoA derived from fatty acid metabolism (Figure 1).

As precursors for flavonoid biosynthesis, 4-coumaroyl CoA, and malonyl CoA are converted to naringenin chalcone by chalcone synthase (CHS) and to dihydrokaempferol by chalcone isomerase (CHI) and flavanone 3-hydroxylase (F3H). Dihydroflavanol 4-reductase (DFR) and anthocyanidin synthase (ANS) are two enzymes that are required to convert dihydrokaempferol to pelargonidin, a pink anthocyanidin. The B-ring of dihydrokaempferol is further hydroxylated either to cyanidin by flavonoid 3′-hydroxylase (F3’H) or to delphinidin by flavonoid 3’5’-hydroxylase (F3’5’H) (Figure 1). Delphinidin-based anthocyanins do not accumulate in chrysanthemum due to the absence of genes encoding F3′5′H [34]. Therefore, only cyanidin-based anthocyanins are reported to accumulate in chrysanthemum ray petals [8]. Several studies have reported that the accumulation of cyanidin, pelargonidin, and delphinidin generally imparts pink to red-purple [43], orange to red [44], and purple to blue [45] respectively.

## 5. Genes Involved in Anthocyanin Biosynthesis

The pattern of petal pigmentation is determined by the expression levels of anthocyanin biosynthetic genes [41]. Mutations that arise either in the regulatory gene or in the biosynthetic gene, may interfere with the interaction between these genes [46]. Structural genes that have been reported to cause pigmentation in flowers include *CHS*, *F3H*, *F3′H*, *DFR*, and *ANS*. In chrysanthemum, seven structural genes, such as *CHS*, *F3H*, *F3′H*, *DFR*, *ANS*, *3GT*, and *3MT* have been identified as key genes responsible for anthocyanin biosynthesis, along with other transcription factors that are considered as major contributors [32,47]. *CmCHS*, *CmCHI*, and *CmF3′H* are highly expressed during early developmental stages, whereas, *CmF3H*, *CmDFR*, *CmANS*, and *CmUFGT* are expressed in a coordinative manner throughout all stages of ray floret development [48]. The pattern of *CmCHI* expression was found to be similar among different cultivars with different anthocyanin contents [49,50]. The high expression levels of *CmDFR* revealed a correlation between anthocyanin accumulation and pink- and white-colored chrysanthemum [32]. Similarly, *3GT* expression was found to be markedly increased in pink flowered chrysanthemum cultivars, suggesting that it contributes to anthocyanin pigmentation [32]. Taken together, these results showed that the transcript levels of biosynthetic genes are consistent with anthocyanin biosynthesis in the ray florets of chrysanthemum.

## 6. Regulation of Anthocyanin Biosynthesis by Transcription Factors

The regulatory mechanisms controlling anthocyanin biosynthesis have been well studied [51]. Members of three protein families, including R2R3MYB transcription factors (TFs), basic helix-loop-helix (bHLH) TFs, and WD40 Repeat Proteins (WDR) interact to form a ternary MBW complex, which subsequently regulates the expression of anthocyanin biosynthetic genes [51,52]. The R3 domain of R2R3 MYB interacts with the MIR region of bHLH, whereas the WDR interacts with a bHLH TF as a docking platform [53]. Other such interactions include *AN2* (MYB) and *AN1* (bHLH) in petunia flowers [54], *MYB10* (MYB) and *MYC1* (bHLH) in *Gerbera hybrida* flowers [55], and *MYB12* and *bHLH2* in lily flowers [56,57]. bHLH has a broad range of biological functions apart from anthocyanin biosynthesis and is therefore expressed in various other cell types where anthocyanins are not produced. However, R2R3 MYB TFs directly regulate the spatio-temporal expression of anthocyanin biosynthetic genes. MYB TFs determine the amount of anthocyanin produced by specific cells and hence, variation in the color intensity and pattern of the flower [34]. Recent studies reported that repressor genes, including R3MYBs such as *AtMYBL2*, and R2R3 MYB such as *AtMYB44* and *PhMYB27*, negatively regulate anthocyanin biosynthesis by interfering with MBW complex formation [58,59].

**Figure 2:**
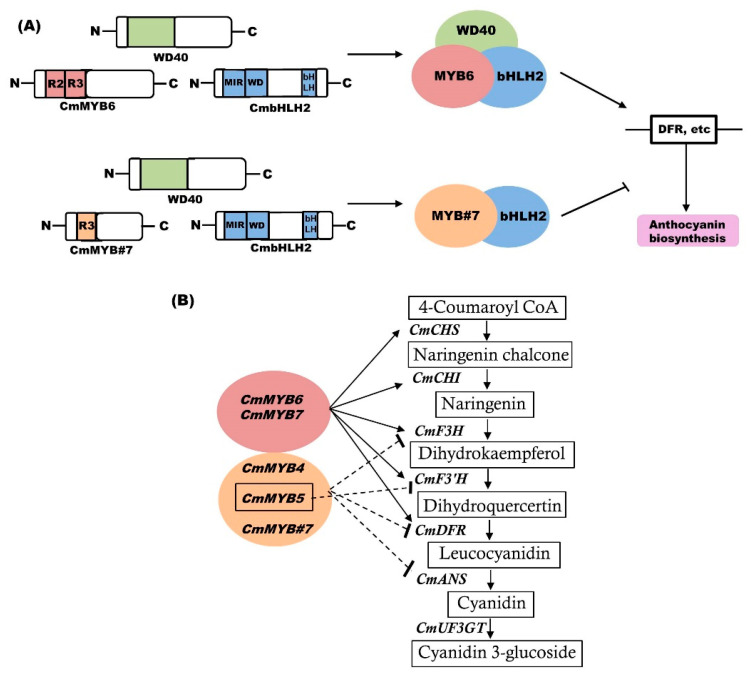
MYB-bHLH-WD40 (MBW) complex for the regulation of anthocyanin biosynthesis. (**A**) Schematic model to depict the regulatory mechanism of the MBW complex. bHLH proteins interacts with MYB transcription factors (TFs) and WD40 to form a ternary complex, thereby regulating structural genes. (i) Activation of anthocyanin biosynthesis regulation by MYB-activators. (ii) Repression of anthocyanin biosynthesis by MYB-repressors, which compete with MYB-activators for bHLH. Arrows (‘→’) indicate activation, and blocked lines (‘—|’) indicate repression. (**B**) Representative MYBs for the regulation of anthocyanin biosynthesis. *CmMYB6* and *CmMYB7* are positive regulators of anthocyanin biosynthesis. In contrast, *CmMYB4*, *CmMYB5,* and *CmMYB#7* are repressors of anthocyanin biosynthesis. The arrow indicates activation and the dotted blocked arrow indicates repression (modified from [66].

The bHLH proteins belong to a superfamily of TFs containing a basic region with 15–17 amino acids required for DNA binding and a helix-loop-helix domain essential for the formation of homo and heterodimers [60]. A bHLH member was reported to be involved in the regulation of anthocyanin biosynthesis in Dahlia and Asiatic hybrid lily [56,61]. In *Phalaenopsis*, the endogenous bHLH member, interaction between *PebHLH1-3* and PeMYBs was shown to be responsible for the pigmentation patterning in flowers [62]. Recently, Lim et al. (2017) revealed that *RsTT8* functions in anthocyanin and proanthocyanidin biosynthesis through an interaction with *RsMYB1* [63]. Based on clustering analysis, three CmMYBs and one CmbHLH were identified as candidate TFs for anthocyanin biosynthesis in chrysanthemum [47]. Liu et al. (2013) [64] found that endogenous bHLH proteins in conjunction with *MYB1* are involved in anthocyanin biosynthesis. Based on this, further studies showed that the level of *CmbHLH2* was positively correlated with anthocyanin content in the various colored flowers of chrysanthemum. In a simultaneous expression assay with *CmMYB6*, *CmbHLH2* significantly upregulated the *CmDFR* promoter and triggered anthocyanin biosynthesis, suggesting that *CmbHLH2* is crucial for anthocyanin biosynthesis in chrysanthemum [65]. A representative model depicting the regulatory mechanism by TFs in chrysanthemum is shown in Figure 2A.

MYB proteins represent a TF family in plants. The N-terminal domain of MYB is essential for DNA binding, while the C-terminal domain is involved in the regulatory activity of the protein [67]. MYBs are the most specific and crucial regulators of anthocyanin biosynthesis among the MBW complex [68]. Role of MYBs has been reported in various plants, including *GMYB10* from *Gerbera hybrida*, *LhMYB12-Lat* from *Asiatic hybrid lily*, and *PhAN2* from *Petunia hybrida* [69,70,71]. In chrysanthemum, three candidate CmMYBs for anthocyanin biosynthesis have been identified by cluster analysis [47]. Liu et al. (2015) identified four MYBs, including *CmMYB3*, *CmMYB4*, *CmMYB5*, and *CmMYB6* from chrysanthemum ‘Amadea’ [72]. Among those, the expression of *CmMYB3* and *CmMYB6* was correlated with anthocyanin accumulation. Additionally, *CmMYB6* alone activated the *CmDFR* gene and induced anthocyanin biosynthesis in tobacco leaves when transiently co-expressed with a previously characterized anthocyanin regulator, *MrbHLH1* [72]. Transgenic chrysanthemum expressing *RsMYB1* presented enhanced accumulation due to the upregulation of *CmF3H*, *CmDFR*, and *CmANS* [73]. Recently, R3 MYB, *CmMYB#7* was identified as a passive repressor of anthocyanin biosynthesis [74]. *CmMYB#7* competes with *CmMYB6* for the interaction with *CmbHLH2*, and inhibits the active MBW complex, resulting in the suppression of structural gene expression [66]. Hong et al. (2019) showed that *CmMYB6* and *CmMYB7* positively regulate anthocyanin biosynthesis genes, whereas *CmMYB4* and *CmMYB5* are negative regulators [66]. It has been hypothesized that the control of *CmCHS*, *CmCHI*, *CmF3H*, *CmDFR*, and *CmANS* is coordinated by multiple regulators, whereas *CmF3’H* might be solely regulated by other TFs, such as *CmMYB5* [66]. The results of Xiang et al. suggested that four CmMYBs might interact with bHLH TF(s) and consequently activate or repress the transcript levels of anthocyanin biosynthetic genes [74]. However, it is unclear whether these four CmMYBs interact with the same bHLH proteins as *CmMYB#7* (Figure 2B). Hence, protein-protein interaction studies among these CmMYBs, and *CmbHLH2* [74] and *CmbHLH24* [47] will provide insight into the mechanisms of anthocyanin biosynthesis in chrysanthemum.

## 7. External Factors Regulating Anthocyanin Biosynthesis and Accumulation

Anthocyanin biosynthesis is predominantly influenced by various physical and chemical factors. Therefore, this section discusses regulatory factors that have been reported to influence anthocyanin biosynthesis and thus, floral color.

### 7.1. Physical Factors

Various physical factors, such as temperature, light intensity and quality, water content, nutrients, and minerals have been identified to have a major influence on the accumulation of anthocyanin content in flower petals.

#### 7.1.1. Light Intensity and Quality

Light intensity is one of the key factors that can influence anthocyanin biosynthesis [75]. Early studies demonstrated the light-induced accumulation of anthocyanins in various plants, such as *Arabidopsis* and *Petunia hybrida* [76,77]. Furthermore, the absence of light or weak light intensity is likely to suppress the expression of anthocyanin biosynthetic genes, resulting in a significant decrease in anthocyanin content in the flowers of various plants [78,79,80]. In chrysanthemum petals, shading was found to result in a significant decrease in anthocyanin accumulation, resulting in an almost white-colored flower [81]. Hong et al. reported that capitulum and foliage are the key organs that respond to light for anthocyanin accumulation in chrysanthemum flowers [81]. Recently, an in-depth review of molecular studies on chrysanthemum ray petals noted that transcriptional activators, such as *MYB5-1*, *MYB6*, *MYB7-1*, *HY5*, *COP1*, and *bHLH24*, are key genes involved in the light-induced accumulation of anthocyanin, in addition to anthocyanin biosynthetic genes in chrysanthemum ray petals [8]. More recently, a transcriptome analysis of *C. morifolium* ‘Jinbeidahong’ under short day (SD) and natural day (ND) light conditions, in which floral color was lighter under SD compared to ND, found that anthocyanin biosynthesis was tightly regulated by photo-period. The *HY5-1*, *HY5-2*, *HY5-3*, *HY5-4*, *CHI1*, and *3GT1* genes plays a significant role in the induction of anthocyanin biosynthesis under photo-periodic lighting [82].

The importance of light quality in the regulation of anthocyanin accumulation in flowers has been described in various plants, including petunia and rose [83,84]. Early findings showed that petunia has three photoreceptors that are involved in the regulation of anthocyanin synthesis [80]. In *Arabidopsis*, dihydroflavonol 4-reductase (DFR) is induced by red light, and chalcone synthase (CHS) is induced by blue light, whereas in gerbera, blue light is more effective at inducing anthocyanin accumulation [78,79]. A study by Ouzounis and co-authors showed that an increased blue light component induced flavonoid synthesis in chrysanthemum [84]. The levels of total flavonoids were found to be higher under white light in chrysanthemum leaves [85].

#### 7.1.2. Temperature

Temperature is another physical factor that influences the expression of floral color in ray petals. Higher temperature was shown to cause a significant reduction in anthocyanin accumulation in various flowers, including lily [86], rose [87], petunia [88], and carnation [89], suggesting that higher temperature poses a major problem for floral color during the summer season in temperate regions and throughout the year in tropical zones. In particular, poor pigmentation has been observed at higher temperatures (30 °C) in different genotypes of chrysanthemum [90]. Additionally, the degree of color variation was found to differ among chrysanthemum genotypes at 30 °C, although no color change occurred in some genotypes. Studies by Nozaki et al. and Huh et al. reported that the pale pigmentation in pink-flowered genotypes of chrysanthemum was mainly due to a reduction of two anthocyanins (Cy 3-6’’-MMG and Cy 3-3’’,6’’-DMG) under high temperature [91,92]. The total anthocyanin content was found to be considerably higher at 20 °C compared with 30 °C in chrysanthemum petals [93]. Expression of anthocyanin biosynthesis-related genes, including *CHS1*, *CHS2*, *CHI*, *F3H2*, *C3′H*, *DFR1*, *DFR2*, and *ANS* was downregulated in ray petals when the plants were exposed to 30 °C.

### 7.2. Chemical Factors

Several reports had detailed the influence of environmental pH on the color intensity and pigmentation of flower petals [94,95]. Various studies have also shown that acidic pH has a positive effect on anthocyanin accumulation in rose and petunia [94,96]. An interesting study by Zaho and co-authors reported that the pH of petals was highly influenced by the pH of irrigated water in herbaceous *peony* [97]. Briefly, at pH 4.0, the pH of petals increased and the anthocyanin content reduced significantly, and herbaceous peony exhibited a lighter floral color. Increased expression of the pH-regulating gene vacuolar Na^+^/H^+^ antiporter 1 (*NHX1*), and decreased expression of the anthocyanin biosynthesis *DFR* gene, have been shown to be responsible for the reduced color in peony [97]. Furthermore, in chrysanthemum, vacuolar pH is a key factor that modulates the biosynthesis and quantity of anthocyanin pigments. The development of blue color in flowers has been well studied, and one of the mechanisms that achieves this is the modification of vacuolar pH [98,99]. Blue-colored ray florets of transgenic chrysanthemum generated by expressing the anthocyanin, delphinidin 3-*O*(6′′-*O*-malonyl) glucoside-3′,5′-di-*O*-glucoside presented a violet color under mildly acidic pH and a blue color only under co-pigmentation with other flavone glucosides in planta [40]. Recently, Rusishvili et al. combined computer simulations and photo absorption experiments of cyanidin-3-*O*-glucoside in water solution and elucidated the molecular mechanisms of color expression in anthocyanins within the pH range of 1–9 [100]. This study demonstrated that, in addition to the charge state of the molecule, pH also affects internal distortions in the chromophore, which influence their degree of conjugation, modulating the optical gap, and thus, the color.

Numerous studies have investigated the effects of plant hormones on the determination of floral color. For example, the transportation of gibberellins from the anther to the petal were reported to significantly upregulate the expression of anthocyanin biosynthesis genes [101]. Growth retardants can efficiently alter flower color. Application of prohexadione-calcium (Pro-Ca) changed the floral color from red to pale pink to white color in China rose [102]. Another study on daminozide, a chemical inhibitor of gibberellin biosynthesis, reduced floral coloration [103]. Menhenett et al. observed that the application of daminozide on dark pink chrysanthemum cultivar abrogated pigment production [104]. Subsequently, the potential underlying biochemical catalysis of daminozide revealed that the loss of red pigmentation in the ray petals of chrysanthemum was highly correlated with enzymatic inhibition of anthocyanidin synthase, resulting in reduced anthocyanidin content [105].

In addition to the above, anthocyanin content and accumulation are also affected by various other factors. For example, prolonged water stress caused flowers to darken in oriental hybrid lily [86], whereas in the leaves of *Chrysanthemum morifolium* cultivars, anthocyanin content increased with increasing water stress [106]. Gamma irradiation [107] and ion beam radiation [108] were found to influence anthocyanin accumulation in chrysanthemum flowers. Recently, Ryu et al. conducted a comparative study in Gamma-irradiated mutant *Chrysanthemum morifolium* cultivars and observed that the mutant cultivars producing purple to pink ray petals, accumulated higher amounts of anthocyanins compared with original cultivars [109]. Conversely, mineral nutrients are widely known to regulate floral color. Phosphorous deficiency has been associated with the increased synthesis of anthocyanins and is responsible for a darker coloration [110]. This may be because phosphorous deficiency reduces the synthesis of carbohydrates, resulting in an increased amount of soluble sugars and anthocyanins [111]. The presence of potassium has a favorable effect on floral color [112]. However, the effect of the same potassium differs among various colored varieties. Spraying potassium was shown to improve the floral color of orange and red varieties, but did not affect yellow varieties in lily. The mechanism underlying the change in pigmentation remains unclear [113].

## 8. Breeding for Anthocyanins

Plant breeding is the purposeful manipulation of plant species to develop desired phenotypes and genotypes with specific traits. Several breeding strategies have been employed for floral color, floral shape, plant architecture, disease resistance, and stress tolerance. Various tools are used to improve plant traits, including cross breeding, mutational breeding, and breeding by genetic modification. A large number of cultivars have been developed through cross-breeding and mutational breeding, which apply to a limited number of traits. Additionally, transgenic technologies have been applied to a variety of ornamental plants to improve desired traits by engineering the genomes [114]. The most fascinating blue-colored flowers in roses and chrysanthemums were made possible by the use of powerful transgenic technology in combination with genetic and biochemical analyses. This would not have been possible using conventional breeding techniques, especially in roses and chrysanthemums as they lack the delphinidin pathway [40,45]. Methods of breeding that are employed to modify floral color are discussed in the following sections.

### 8.1. Crossbreeding

Crossbreeding is a powerful classical tool that has been practiced for decades to generate attractive cultivars, with new traits in the majority of crops. As chrysanthemums are highly heterozygous, conventional crossbreeding between two parents possessing contrasting traits remains the most effective way of breeding new cultivars [5]. As genetic differences in parents and hybrid performance are positively correlated, the selection of parents with genetic variation is a key factor for successful breeding [115]. Intra- and inter-specific hybridization have been used in chrysanthemum for many traits, including plant type [116], stress resistance [117], and flowering time [116,118]. Numerous cultivars have been developed by crossbreeding for floral color in chrysanthemum. For example, an inter-specific cross between hexaploid *C. weyrichii (Maxim.) Tzvelv*. and *C. grandiflora Tzvelv.* resulted in a new garden chrysanthemum cultivar ‘Lavender Daisy’ with a new floral color for the Mammoth series, exhibiting dark purple ray florets and golden disc florets [119]. Mammoth ‘Dark Bronze Daisy’, with novel dark bronze daisy flowers and gold disk florets, was developed by an inter-specific cross between *C. weyrichii (Maxim.) Miyabe* ‘Pink Bomb’ × *C. grandiflorum Tzvelv.* ‘Adorn’ [120]. 

**Figure 3:**
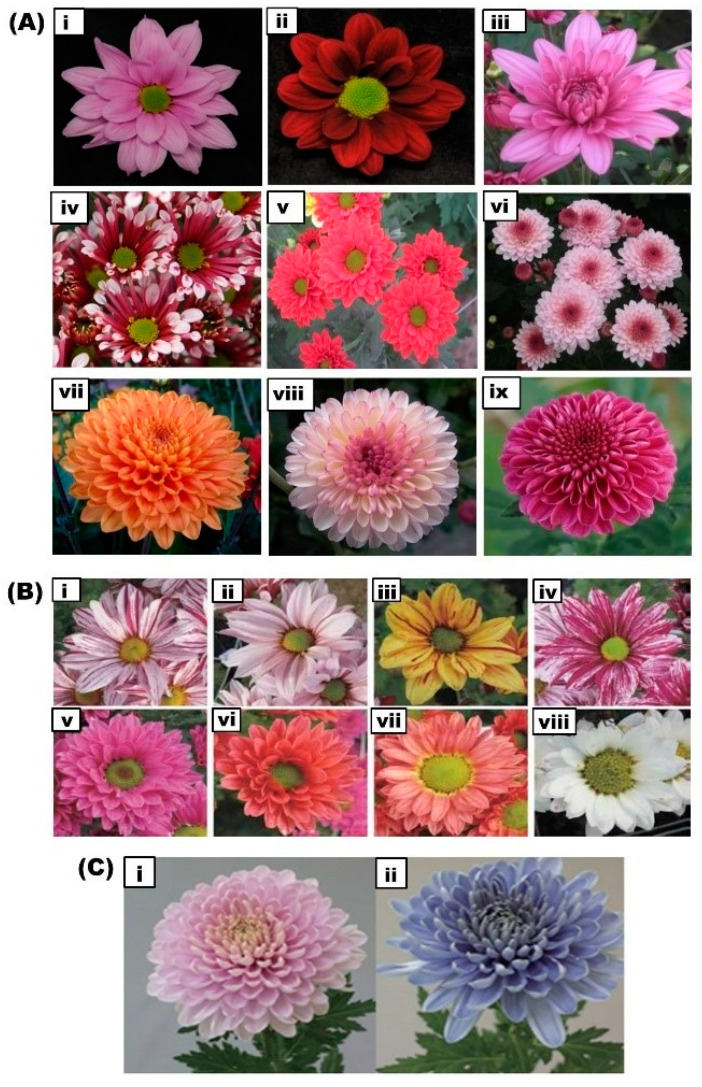
Representative images showing the modification of floral color in chrysanthemum by various breeding methods. (**A**) Diverse and vibrant colored flowers expressing anthocyanins in chrysanthemum cultivars developed by our work through cross breeding at (National Institute of Horticultural and Herbal Sciences (NIHHS), RDA, Korea. (**i**): Glory pink; (**ii**): Red marble; (**iii**): Donna pink; (**iv**): Purple cone; (**v**): Princeling; (**vi**): Cutie pink; (**vii**): Orange pangpang; (**viii**): Pink pangpang: and (**ix**): Purple pangpang. (**B**) Modification of floral color by mutation breeding. A range of floral colored mutants presenting various colors were generated by gamma-irradiation in two chrysanthemum cultivars ‘Noble wine’ and ‘Pinky’. (**i**): Original floral color of ‘Noble wine’; (**ii**–**iv**): gamma-irradiated mutants of ‘Noble wine’ showing varied floral colors. (**v**): Original floral color of ‘Pinky’; and (**vi**–**viii**): gamma-irradiated mutant floral colors of ‘Pinky’ [107]. (**C**) Generation of blue-colored chrysanthemum flowers by metabolic engineering. (**i**): wild-type red-purple colored flower; (**ii**): transgenic blue-colored flower generated in combination with the overexpression of *CamF3’5’H* and *CtUGT,* and co-pigmentation [40].

In a recent study, inter-specific crossability of transgenic blue chrysanthemum developed by Noda et al. [40] with the wild species *Chrysanthemum japonense* var. japonense was checked [121]. This study demonstrated the heritability of the transgene and confirmed the transmission of the transgene to interspecific progeny. Furthermore, the progeny also accumulated anthocyanins that were specific to the parent plant. This provides basic information that may be used to prevent transgene flow to native chrysanthemum species in order to commercialize the blue-hued varieties [121]. Intriguingly, our work on the breeding of new chrysanthemum cultivars for novel floral colors using inter- and intra-specific breeding has resulted in a large number of cultivars exhibiting alluring flower hues. Flower images of some of the chrysanthemum cultivars developed from our work are shown in Figure 3A [122,123].

### 8.2. Molecular Breeding

Floral color is one of the most widely studied traits at the genetic level, and various studies have characterized the mutations responsible for floral color variations in plants [124]. Studies have revealed that variations in floral color involve the common genetic route, which indicates optimal methods to modify floral color [125]. To date, the chrysanthemum breeding program has resulted in the development of various cultivars. However, further advances in conventional morphology-based selection are needed for molecular marker-assisted breeding. These molecular breeding studies require the identification of trait-linked molecular markers from genetic, molecular, and genomic studies. However, the reproductive, genomic, and genetic makeup of chrysanthemum, including self-incompatibility, inbreeding depression, allohexaploid, heterozygosity, genome duplications, and large genome size, significantly hinder these studies. Molecular breeding, popularly known as marker-assisted selection (MAS), enables the identification of markers associated with plant traits, which also indirectly select the phenotype of the plant. The linkage map facilitates the identification of genomic regions associated with the desirable traits, thereby providing a base for MAS-breeding. Furthermore, quantitative trait loci (QTL) mapping identifies the genes that control a trait based on linkage mapping [126]. However, MAS-breeding in polyploids, such as chrysanthemum, is progressing at a slower pace due to various technical and scientific limitations [127]. This is because the linkage mapping and QTL analysis for DNA marker development require specialized statistical methods in order to estimate recombination frequency and achieve QTL detection [128]. Nevertheless, numerous genetic studies have successfully constructed genetic linkage maps and identified molecular markers, including amplified fragment length polymorphism (AFLP), random amplified polymorphic DNA (RAPD), sequence-related amplified polymorphism (SRAP), simple sequence repeat (SSR) markers, and single-nucleotide polymorphism (SNP) markers associated with important plant and flower traits in chrysanthemum [129]. Advances in sequencing technologies have provided whole genome sequence information for the diploid wild chrysanthemum species, *Chrysanthemum nankingense* [130] and *Chrysanthemum seticuspe* [131]. This contributes to further studies exploring the connection between genotype and phenotype, and will help to overcome some of the barriers for molecular breeding in chrysanthemum. QTL detection in chrysanthemum has been achieved using sophisticated methods to handle its genome complexity. Van Geest et al. reported the first integrated ultra-dense linkage map in a hexaploid species with polysomic inheritance in chrysanthemum, which identified some of the QTLs for floral color along with other floral traits, such as flowering time, ray floret number, and disc florets de-greening [132]. Two regions, CLG5 and 7, were found to be significantly associated with floral color and one region, CLG9, was slightly associated with floral color. Analysis of variance of the interaction between the associated alleles identified a highly significant interaction, suggesting that both alleles are required to obtain pink-colored flowers [132]. This study represented a breakthrough for further linkage analyses in chrysanthemum. Genome-wide association studies (GWAS) are an effective tool that connects the phenotype and genotype based on the available linkage disequilibrium [133]. In chrysanthemum, considerable progress has been made in recent years to identify favorable alleles by GWAS for traits such as waterlogging tolerance [134], aphid resistance [135], drought tolerance [136], plant architecture traits, and inflorescence traits [137] based on SRAP, start codon-targeted (SCoT), and SSR marker loci. Chong et al. first reported a GWAS for four floral traits of floral color, floral shape, ray floret type, and cultivated type, and identified seven SNPs within six genes that are predictive of only floral shape, ray floret type, and cultivated type [138]. However, these SNPs did not show any association with floral color. Shumitomo and co-authors reported the first simple GWAS-based marker developing system in autohexaploid species, such as chrysanthemum [128]. Their study aimed to verify whether DNA markers for carotenoid pigmentation, which determines ray petal color, could be effectively developed using GWAS in autohexaploid chrysanthemum. Because carotenoid pigmentation in the petal is controlled by the dominant allele of the inhibitor, and a casual gene *CmCCD4a* is responsible for carotenoid cleavage in ray petals, a parent ‘Ariesu’ was identified as duplex for the carotenoid cleavage allele. Hence, SNP markers developed for each allele have demonstrated a perfect association with carotenoid cleavage in ray petals and with the absence or presence of the genes *CmCCD4a-1* and *CmCCD4a-5* in the F1 population, indicating the effectiveness of GWAS method. Together, these studies represent ground-breaking advances towards the integration of phenotypic and genotypic information, and for further advancement in the breeding of chrysanthemum for desirable traits like floral color.

### 8.3. Mutation Breeding

Although crossbreeding is a powerful tool for the development of cultivars, it is quite complicated in heterologous ornamental plants. In this case, mutation breeding is suitable for modifying a few traits in an elite cultivar without affecting the existing traits. This method is highly efficient for creating sterile interspecific hybrids [139], native ornamentals with a limited gene pool in given species [140], and plants with longer juvenile periods [141]. Usually, ultraviolet light, chemical mutagens, and ionizing radiation, such as X-rays and gamma rays, are used to induce a wide spectrum of mutations in ornamental plants. This induced mutagenesis is an established method and has been successfully exploited for the development of diverse novel ornamental varieties in various crops. The induction of mutations in the biosynthetic pathways of structural and regulatory genes has resulted in changes in floral color in various plants, including *Phalaenopsis* and *Gentian* plants [142,143,144]. Mutations in the early steps of the anthocyanin biosynthesis pathway result in white flowers, whereas mutations in the later parts of the pathway lead to the generation of different colored flowers due to the accumulation of particular anthocyanins [145]. Recently, heavy-ion or ion beams have been used to generate new varieties in carnation, rose, and chrysanthemum [146]. Mutation breeding has been successfully employed for decades in chrysanthemum to develop novel floral colors using X-ray irradiation [147,148] and gamma irradiation [149,150,151] through the modification of the anthocyanin biosynthesis pathway (Figure 3B). For example, six mutant varieties have been generated by ion-beam radiation in chrysanthemum for different variations in floral color, including a complex with light yellow and pink; a complex with light yellow and light pink; a complex with light pink and yellowish-orange; a complex with light pink and bright orange-yellow; and a variation with light orange on the adaxial side and dark yellow-orange on the abaxial side [152]. There are some major limitations to mutation breeding; for example, these mutations are not programmable to specific locations, and the chimeric status of each cell possesses different mutations. However, a combination of irradiation and tissue culture is used to resolve the chimeras in chrysanthemum [153].

### 8.4. Genome Editing

Genome editing enables the induction of mutations in a targeted genomic region and has played a substantial role in plant functional genomics and biotechnology [114]. Due to the generation of precise mutations in the targeted sequence, it is considered to be more effective than conventional mutation breeding. This breakthrough technology has been adopted for various crops [124]. Unlike transgenic technology, genome editing does not require the transgene to be integrated with the genome, and hence an integrated transgene could be segregated in the progeny. The clustered regularly interspaced short palindromic repeats (CRISPR)-associated protein 9 (Cas9) system, is the most widely used genome editing system [154]. Cas9 is a nuclease that can cleave the double-stranded DNA of the target sequence, and insertion/deletion mutations of variable length can be introduced into the target sequence during the repair of the Cas9-induced double-stranded breaks [114].

Application of the CRISPR-Cas9 system to ornamental plants has been limited due to a lack of whole genome information to identify the target sequence and transformation system. To date, only a few successful genome-edited ornamental plants on floral traits have been reported. *Petunia* has been edited with CRISPR-Cas9 targeting various traits, including an albino phenotype [155], flower longevity [156], deficiency in nitrate assimilation [157], and self-incompatibility [158]. The *LpPDS* gene in two *Lilium* species was successfully edited resulting in albino, pale yellow, and albino-green chimeric mutants [159]. Multiple MADS genes have been targeted in *Phalaenopsis* for flower initiation and development [160]. CRISPR-Cas9 has been applied to various ornamental plants for floral color. The flavonoid biosynthesis gene (*F3’H*) was edited in *Torenia fournieri*, presenting a pale blue floral color [161]. A knock-out mutant of the *CCD4* gene was generated through genome editing, which harbored the ability to degrade carotenoid, and presented a yellow pigment due to its accumulation in both *Japanese morning glory* and *Ipomoea nil* [162,163]. However, the generation of genome-edited plants with a simultaneous mutation in polyploid plants is complicated, because the double-strand break and repair events occur at each target site independently. Hence, the higher the number of target sites, the lower the probability of mutation in these plants [114]. Whole-genome information is not yet available for chrysanthemum due to its large genome and hexaploidy. However, Kishi-Kaboshi et al. first reported that disruption of fluorescence protein in chrysanthemum resulted in decreased fluorescence in flowers [114]. A transgenic chrysanthemum carrying the yellowish-green fluorescent protein (*CpYGFP*) gene was used for genome editing and was mutated by two sgRNAs targeted *CpYGFP* at different positions. The resultant CRISPR-CpYGFP-edited flowers presented decreased fluorescence compared to the normal CpYGFP flowers [114]. Hence, this result provides a basis and scope for further trials of genome editing in complex polyploid chrysanthemum plants. A limitation of genome editing in vegetatively propagated polyploid plants such as chrysanthemum and potato that cannot segregate the transgene, is that it is not feasible to introduce simultaneous mutations without transgene integration. However, CRISPR-Cas9 genome editing remains a revolutionary and promising technology for crop breeding in chrysanthemum for beneficial traits such as floral color.

### 8.5. Transgenics for the Modification of Anthocyanin Accumulation

In addition to classical breeding, metabolic engineering of anthocyanins is used to develop commercially desirable floral colors. Two strategies are employed to modify floral color. One approach is to overexpress the gene at the rate-limiting step or to block a gene in the competitive pathway. The second approach is to use TFs to alter anthocyanin accumulation [164]. Genetic modifications have been successfully applied to change the floral color in various plants, including rose and carnation [45,165]. Kee et al. developed a transgenic chrysanthemum overexpressing *RsMYB1*, in which the expression of three anthocyanin biosynthetic genes, such as *F3H*, *DFR*, and *ANS*, was elevated. However, there was no visual enhancement of anthocyanin in the transgenic plants [73]. Members of the family Asteraceae, such as *cineraria* and *African daisy*, can accumulate delphinidin-based anthocyanins in their flowers due to the activity of *F3′5′H* [166]. However, the *Chrysanthemum* genus in Asteraceae does not contain *F3′5′H* activity and hence is unable to synthesize delphinidin-based anthocyanins. Therefore, it is difficult to obtain blue flowers by cross-breeding within the genus. Tanaka et al. reported that the genes encoding F3′5′H exist in various plants, which serve as a powerful tool for delphinidin-based anthocyanin pigment accumulation [167]. To generate a blue flower in chrysanthemum, He et al. attempted to reconstruct the delphinidin pathway by overexpressing the *F3′5′H* gene from *Senecio cruentus* and downregulating the *F3′H* gene to block cyanidin synthesis. However, the transgenic chrysanthemum presented bright red petals due to the increased level of cyanidins [48]. Genetic engineering of delphinidin production was achieved via transformation with a chimeric pansy *F3’5’H* gene under the control of floral-specific promoters, resulting in violet/blue-colored petals in chrysanthemum flowers [168]. Similarly, delphinidin production was manipulated by combining a chrysanthemum *F3H* promoter-driven alcohol dehydrogenase (*ADH*) translational enhancer-fused *Campanula F3′5′H*, leading to the formation of blue/violet flowers in chrysanthemum [39]. Noda et al. (2017) successfully produced true blue-colored chrysanthemum flowers by introducing the butterfly pea uridine diphosphate (UDP)–glucose: anthocyanin 3′,5′-*O*-glucosyltransferase gene, in addition to expression of the Canterbury bells *F3′5′H* gene [40] (Figure 3C). These studies have therefore established a powerful basis for the molecular breeding of delphinidin-producing chrysanthemums.

## 9. Absence of Floral Anthocyanins

A lack of either anthocyanins or carotenoids usually produces white petals. However, the majority of white flowers contain a subgroup of flavonoids, such as flavonols and flavanones. Although flavonols and flavanones are colorless pigments, the petals that accumulate a significant amount of these pigments exhibit either a cream or ivory color [32]. A lack of these flavonoids results in the development of pure white petals in some cultivars of snapdragon and carnation [169,170]. In some chrysanthemum cultivars, anthocyanins could not be detected in the petals of white flowers [171]. An absence of anthocyanins occurs due to abrogation of the anthocyanin biosynthesis pathway, because of mutations in the biosynthesis genes or TFs in the pathway [8]. Expression of the biosynthetic genes *DFR* and *3GT* was found to be significantly lower in white petals compared to pink petals in chrysanthemum cultivars [32]. The mechanism for the suppressed gene expression in white petals needs to be further elucidated.

## 10. Conclusions and Future Prospects

Floral color is a major breeding target, because it determines the commercial value of ornamental plants. Anthocyanins are the important pigments that can attribute the petal color in flowers. In nature, chrysanthemums accumulate cyanidin-based anthocyanins, and produce fair pinkish to red to purple colored flowers. Variation in the number of anthocyanins changes the intensity of floral color and shifts in the pigment composition or multiple pigment combinations alter the floral hue. Several physical and chemical factors, as well as genetic and transcriptional regulators, influence anthocyanin accumulation and petal color. Research on anthocyanin biosynthesis and accumulation has progressed substantially in chrysanthemum in recent years. Genes involved in the anthocyanin biosynthesis pathway and their expression profiles have been elucidated in chrysanthemum. These insights have enabled the successful development of blue-colored flowers in chrysanthemum, even though chrysanthemum lacks delphinidin-based anthocyanins in nature. Due to the importance of environmental and product safety, the future development of technology to prevent possible gene flow is essential for commercializing these transgenic chrysanthemums. Furthermore, studies on the upstream regulatory mechanisms that control anthocyanin accumulation are needed. Hence, an understanding of the genetic and molecular mechanisms that control the organ and cellular- specific accumulation of anthocyanins is required. The transcriptome [172,173] and genome [131] of chrysanthemum species provide a basis for the identification of genes of interest. Nevertheless, studies on the genetic mechanisms that control anthocyanin biosynthesis are challenging and are still lagging behind in chrysanthemum. Elucidating the genetic control of anthocyanin biosynthesis enables the development of molecular markers for these traits. Although MAS has been extensively developed in chrysanthemum, further development of a large number of molecular markers would provide a basis for determining the alleles of a particular gene, which would be a useful tool for breeders to achieve the desired color for the flower. This could be achieved by further research focusing on QTL mapping and GWAS, which can dissect the complex genetic control involved in regulating anthocyanin biosynthesis. Additionally, integration of multiple omics platforms and their application, which serve to identify candidate genes to improve and modify floral color, are still not adequate in chrysanthemum. Furthermore, genome editing by the revolutionary CRISPR/Cas9 tool has huge potential for the functional analysis of traits. In the future, this could represent a breakthrough technology to modify and achieve the target floral color. Overall, this review offers a reference for the promotion of breeding and genetic engineering for the development of novel and diverse hues in flowers.

## Figures and Tables

**Table 1:** Major pigments in plants that impart color to floral petals.

Pigment	Class	Structure	Compound	References
Carotenoids	Carotenes	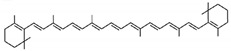	Lycopene, α-carotene, β-carotene, γ-carotene, etc.	[10]
	Xanthophylls	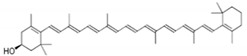	Lutein, zeaxanthin, violaxanthin, neoxanthin, etc.	[10]
Flavanoids	Chalcones	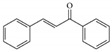	Chalconaringenin, arbutin, phloretin, phloridzin	[11]
	Flavanones	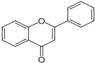	Naringenin, eriodictyol, hesperetin, homoeriodictyol	[11]
	Flavones	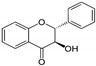	Apigenin, luteolin, tangeritin	[11]
	Flavanonols	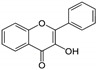	Taxifolin, dihydrokaempferol	[11]
	Flavonols	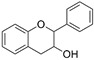	Quercetin, kaempferol, myricetin	[11]
	Flavanols	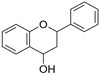	Catechin, epicatechin, apiforol, lueoforol	[11]
	Anthocyanins	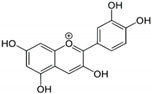	Cyanidin, pelargonidin, delphinidin, malvidin, peonidin, petunidin, etc.	[13]
